# Vaginal colonization with antimicrobial-resistant bacteria among women in labor in central Uganda: prevalence and associated factors

**DOI:** 10.1186/s13756-021-00897-9

**Published:** 2021-02-17

**Authors:** Josephine Tumuhamye, Hans Steinsland, Freddie Bwanga, James K. Tumwine, Grace Ndeezi, David Mukunya, Olive Namugga, Agnes Napyo Kasede, Halvor Sommerfelt, Victoria Nankabirwa

**Affiliations:** 1 Centre for Intervention Science for Maternal and Child Health, Department of Global Public Health and Primary Care, University of Bergen, Bergen, Norway; 2 Centre for International Health, Department of Global Public Health and Primary Care, University of Bergen, Bergen, Norway; 3 Department of Biomedicine, University of Bergen, Bergen, Norway; 4 Department of Medical Microbiology, Makerere University College of Health Sciences, Kampala, Uganda; 5 Department of Paediatric and Child Health, Makerere University College of Health Sciences, Kampala, Uganda; 6 Department of Public Health, Busitema University, Busitema, Uganda; 7 Department of Epidemiology and Biostatistics School of Public Health, Makerere University, Kampala, Uganda

**Keywords:** Antimicrobial resistance, Multidrug resistance, MDR, ESBL, MRSA, MLSB, Carbapenem-resistant bacteria, Vaginal colonization

## Abstract

**Background:**

According to WHO ( CISMAC. Centre for Intervention Science in Maternal and Child health), the antimicrobial resistant bacteria considered to be clinically most important for human health and earmarked for surveillance include extended-spectrum beta-lactamase (ESBL)-producing *Enterobacteriaceae,* carbapenem-resistant bacteria, methicillin-resistant (MRSA) and, macrolide-lincosamide-streptogramin B -resistant vancomycin-resistant (VRSA) *Staphylococcus aureus* and vancomycin-resistant *Enterococcus* (VRE). If these bacteria are carried in the female genital tract, they may be transmitted to the neonate causing local or systemic neonatal infections that can be difficult to treat with conventionally available antimicrobials. In order to develop effective treatment strategies, there is need for updated information about the prevalence of colonization with important antimicrobial-resistant pathogens.

**Objective:**

We sought to estimate the prevalence of vaginal colonization with potentially pathogenic and clinically important AMR bacteria among women in labour in Uganda and to identify factors associated with colonization.

**Methods:**

We conducted a cross-sectional study among HIV-1 and HIV-2 negative women in labour at three primary health care facilities in Uganda. Drug susceptibility testing was done using the disk diffusion method on bacterial isolates cultured from vaginal swabs. We calculated the prevalence of colonization with potentially pathogenic and clinically important AMR bacteria, in addition to multidrug-resistant (MDR) bacteria, defined as bacteria resistant to antibiotics from ≥ 3 antibiotic classes.

**Results:**

We found that 57 of the 1472 enrolled women (3.9% prevalence; 95% Confidence interval [CI] 3.0%, 5.1%) were colonized with ESBL-producing *Enterobacteriaceace,* 27 (1.8%; 95% CI 1.2%, 2.6%) were colonized with carbapenem-resistant *Enterobacteriaceae*, and 85 (5.8%; 95% CI 4.6%, 7.1%) were colonized with MRSA. The prevalence of colonization with MDR bacteria was high (750/1472; 50.9%; 95% CI 48.4%, 53.5%). Women who were ≥ 30 years of age had higher odds of being colonized with MDR bacteria compared to women aged 20–24 years (OR 1.6; 95% CI 1.1, 2.2).

**Conclusion:**

Most of the women included in our study were vaginally colonized with potentially pathogenic MDR and other clinically important AMR bacteria. The high prevalence of colonization with these bacteria is likely to further increase the incidence of difficult-to-treat neonatal sepsis.

## Background

The spread of infections with antimicrobial-resistant bacterial pathogens is a global public health challenge [1]. Pathogens that are responsible for most invasive neonatal infections are often resistant to commonly used antibiotics [2], and many are resistant to antibiotics from several different classes, including many last-resort drugs, which further complicates and limits the possibilities for treatment. Infections with these pathogens are associated with prolonged hospital stays, increased risk of complications and of death [3]. In the World Health Organization’s recently published Global Priority Pathogens List, reducing the burden of infection with pathogenic antimicrobial resistant bacteria has been given priority, and combatting the spread of AMR is also listed as one of the main priorities in the United Nations general assembly’s 2030 agenda for sustainable global health development [4].

The AMR bacteria considered to most importantly threaten neonatal health include extended-spectrum beta-lactamase (ESBL)-producing *Enterobacteriaceae,* carbapenem-resistant bacteria, methicillin-resistant *Staphylococcus aureus* (MRSA), macrolide-lincosamide-streptogramin B (MLSB)-resistant *S. aureus,* vancomycin-resistant *S. aureus* (VRSA), and vancomycin-resistant *Enterococcus* spp*.* (VRE) [5, 6]. The incidence of systemic infections with anaerobes is relatively low among neonates [2, 7, 8].

Many AMR bacteria are multidrug-resistant (MDR), which is commonly defined as being resistant to antibiotics from ≥ 3 different antibiotic classes [9]. Being infected with MDR bacteria tends to complicate or prolong treatment since the causative bacteria are resistant to commonly used antibiotics. Hospitalizations or visits to health clinics, direct contact with livestock and overuse of antibiotics are considered to be the most important risk factors for becoming infected with MDR bacteria and other clinically important AMR bacteria [4].

It is thought that pathogenic bacteria colonizes the birth canal mainly after faecal contamination [10] and are then sometimes transmitted to the baby during labour and delivery [11]. Such transmission is probably one of the main sources of neonatal bacterial infection within the first week of life, particularly if there was prolonged / obstructed labour or premature rupture of membranes (PROM) [12–14]. Having access to relevant antimicrobial resistance data for bacterial pathogens colonizing the birth canal can help clinicians make informed treatment decisions for neonatal bacterial infections, and, thus, improve chances of recovery while reducing the risk of complications and death. The availability of antimicrobial resistance surveillance data on a local level also helps to inform national health policies. There is a shortage of up-to-date data on AMR in sub-Saharan Africa. In addition, there is little knowledge about the extent of and risk factors associated with vaginal colonization with AMR bacteria. Here, we isolated potentially pathogenic bacteria from the birth canal of Ugandan woman in labour, determined the antimicrobial resistance patterns of the isolates, and estimated the prevalence of and identified risk factors associated with colonization with such bacteria.

## Methods

### Study design, setting and participants

We conducted a cross-sectional study between July 2016 and July 2018 at three primary health care facilities in and close to Kampala in central Uganda: Mukono General Hospital (formerly Mukono Health Centre IV), Kawaala Health Centre III, and Kitebi Health Centre III [15]. This study was nested within the Chlorhexidine Trial, which is a randomized controlled assessing whether a single application of 4% chlorhexidine solution on the umbilical cord stump immediately after birth reduces the risk of omphalitis and severe illness [16].

Of the 1658 women in labour who were screened for this study, we recruited 1472 who had already agreed to being enrolled in the above-mentioned Chlorhexidine Trial. The inclusion criteria for the trial were: mother was negative for HIV-1 and HIV-2 and gave birth on a weekday, the newborn weighed > 1.5 kgs, had no severe congenital anomalies, had no obvious signs of umbilical cord stump infection and had no severe illness on the day it was born. We enThe sample size was calculated for the trial but not for the present study. With this sample size we would obtain a very high (0.7% to 2.6%) absolute precision, i.e. the difference between the upper limit and the lower limit of the 95% confidence interval (CI) for prevalence estimates ranging from 2 to 50%.

### Consent and interview

Midwives obtained oral consent to collect a specimen from women in labour. After birth, a study nurse confirmed the verbal consent by obtaining written consent. In the subsequent interview, study nurses collected socio-demographic and clinical information from study participants using Open Data Kit-based standardized questionnaires [17].

### Specimen collection and processing

Trained midwives collected the vaginal swabs during labour. A Regular Rayon sterile swab (Copan Diagnostics Inc., Murrieta, CA) was carefully inserted halfway between the introitus and cervix, avoiding contamination from the cervical mucus. The swab was then gently pressed against the vaginal wall, rotated to collect the specimen, and then removed, carefully avoiding contact with other parts of the body. The swabs were immediately stored in Amies Agar Gel without Charcoal transport medium (Copan Diagnostics Inc.) and transported daily in a cold box holding a temperature of 10–25 °C to MBN Clinical Laboratories Ltd in Kampala, which is a private research and diagnostic laboratory currently undergoing accreditation, where the swabs were immediately processed. We did not culture the specimens anaerobically because of the added cost and effort and because anaerobic bacteremia is uncommon in neonates [2].

### Culture methods

The specimens were streaked onto blood agar containing 5% in-house produced sheep blood and onto MacConkey agar (both Biolab Inc., Budapest, Hungary) and incubated aerobically at 35–37 °C for 18–24 h for isolation of single colonies. The blood agar plates were further incubated for a total of 72 h to enable isolation of slow-growing colonies. From each of the two plates, one representative of each morphologically distinct colony was picked and streaked onto new plates and 1–5 resulting colonies from each of them were pooled in saline solution and subjected to further species determination and classification as described below, before being stored at −80 °C in Brain Heart Infusion broth with 20% glycerol.

### Species identification

Identification of bacterial species was mainly done based on colony morphology, Gram staining and on standard biochemical tests. *S. aureus* was identified with the catalase, slide coagulase, mannitol fermentation, and DNase tests. The bile esculin test was performed to identify *Enterococcus* spp. Putative streptococcal isolates were grouped into different Lancefield groups with the Streptococcal Grouping Kit (Oxoid Ltd., Basingstoke, Hants, UK). Lactose and non-lactose fermenting colonies of gram-negative bacilli were identified based on morphology on MacConkey agar, and the isolates were characterized on the species level by performing standard biochemical tests such as Sulphide Indole Motility (SIM test), gas production, citrate test, urease test and oxidase test [18]. If two isolates from the same specimen were of the same species but had different biochemical characteristics, we included both isolates in the analyses. We considered gram-negative isolates representing *E. coli*, *K. pneumoniae, Citrobacter* spp., *Enterobacter* spp*., Acinetobacter* spp., *K. oxytoca, Pseudomonas* spp., and *Proteus* spp*.* and gram-positive isolates representing *S. aureus, Enterococcus* spp.*,* Group A *Streptococcus*, and Group B *Streptococcus* to be potentially pathogenic bacteria and they were thereby included in the present study.

### Antimicrobial drug susceptibility determination

Antimicrobial drug susceptibility testing of the bacterial isolates was performed using the disk diffusion method as described in the 2017 Performance Standards published by the Clinical Laboratory Standard Institute (CLSI) [19]. We also tested for the antibiotics recommended by the same standard. For gram-positive isolates, we tested against the following antibiotic resistance discs purchased from Biolab Inc.: penicillin (10 µg), trimethoprim-sulfamethoxazole (1.25/23.75 µg), chloramphenicol (30 µg), tetracycline (30 µg), ciprofloxacin (5 µg), gentamicin (10 µg), erythromycin (15 µg), oxacillin (1 µg), vancomycin (30 µg), ceftriaxone (30 µg), and linezolid (30 µg). For Gram-negative isolates, we tested against the following discs: trimethoprim-sulfamethoxazole (1.25/23.75 µg), ciprofloxacin (5 µg), chloramphenicol (30 µg), gentamicin (10 µg), amikacin (10 µg), ampicillin (10 µg), amoxicillin-clavulanic acid (20/10 µg), ceftriaxone (30 µg), cefuroxime (30 µg), ceftazidime (30 µg), tetracycline (30 µg), piperacillin (100 µg), piperacillin-tazobactam (100/10 µg), colistin (10 µg), and imipenem (10 µg). The inhibition zone diameters were measured after incubation at 35–37 °C for 24 h, and we considered an isolate to be resistant (i.e. non-susceptible) if the measurements indicated resistance or intermediate resistance to the given drug.

### ESBL-producing *Enterobacteriaceae* identification

To identify ESBL-producing *Enterobacteriaceae*, we used the combination disk method [20] where a combination disk containing 30 µg ceftazidime and 10 µg clavulanic acid was placed 15 mm from a 30 µg ceftazidime disk on a Mueller–Hinton agar plate. Isolates that had clear zones that were ≥ 5.0 mm larger around the combination disk than around the ceftazidime disk were considered to represent ESBL-producing bacteria.

### Carbapenem-resistant bacteria identification

We considered isolates that were resistant to imipenem to be carbapenem-resistant based on CLSI guidelines.

### MRSA identification

To identify methicillin-resistant *S. aureus* (MRSA) genotypically, we performed multiplex PCR-based identification of MRSA of most *S. aureus* isolates, as described by McClure et al*.* [21]. In this assay, the presence of the *mecA* methicillin resistance gene was used to identify MRSA, while the presence of the gene for the Panton-Valentine Leukocidin (PVL) virulence factor was a marker for community-acquired MRSA [22]. The completed reaction was separated on a 2% agarose gel stained with ethidium bromide, and the amplicons were visualized by using a UV trans-illuminator.

### MLSB-resistant *S. aureus* identification

To identify *S. aureus* isolates that had the macrolide-lincosamide-streptogramin B (MLSB) resistance phenotype, we performed the D-test [23]. In this test, disks with 15 µg erythromycin and with 2 µg clindamycin were placed 15 mm apart. If the isolate was resistant to both erythromycin and clindamycin, the isolate was considered to have a constitutive MLSB resistance phenotype (cMLSB), while if it was resistant to erythromycin and susceptible to clindamycin, but there was a D-shaped inhibition zone around the clindamycin disk, we considered the isolate to have an inducible MLSB resistance phenotype (iMLSB).

### VRE and VRSA identification

We considered *Enterococcus* spp. and *S. aureus* isolates that are resistant to vancomycin to represent VRE and VRSA, respectively.

### MDR bacteria

We used the definition proposed by Magiorakos et al. [9], i.e. that isolates non-susceptible to ≥ 1 antibiotic in ≥ 3 of the antibiotic classes were considered MDR. The antibiotic selection was based on the 2017 Performance Standards from CLSI [19], which differs slightly from other commonly used standards, such as those published by EUCAST [24]. The antibiotic classes and antibiotics (given in parentheses) used for the MDR definition included penicillins (ampicillin, piperacillin, penicillin), penicillins and beta-lactamase inhibitors (amoxicillin-clavulanic acid), antipseudomonal penicillins and beta-lactamase inhibitors (piperacillin-tazobactam), non-extended-spectrum beta-lactams such as second generation cephalosporins (cefuroxime), extended spectrum beta lactams such as third generation cephalosporins (ceftriaxone, ceftazidime), carbapenems (imipenem), fluoroquinolones (ciprofloxacin), phenicols (chloramphenicol), folate pathway inhibitors (Trimethoprim-sulfamethoxazole), aminoglycoside (gentamicin, amikacin), anti-staphylococcal beta lactams (oxacillin), glycopeptides (vancomycin), macrolides (erythromycin), tetracyclines (tetracycline), and oxazolidinones (linezolid). As seen in Tables 1 and 2, we did not test for resistance for a given antibiotic if the species is known to be naturally resistant to the antibiotic.

**Table 1:** Vaginal colonization with antimicrobial drug-resistant potentially pathogenic gram-negative bacteria among study women in labor

Antibiotics^a^	Number of the 1472 study women who were colonized with resistant bacteria (prevalence of colonization/proportion of isolates, in %)							
	*E. coli* (n = 504/508)^c^	*K. pneumoniae* (n = 144/145)	*Enterobacter* spp. (n = 32/32)	*K. oxytoca* (n = 23/23)	*Citrobacter* spp. (n = 107/107)	*Acinetobacter* spp. (n = 32/32)	*Pseudomonas* spp. (n = 3/3)	*Proteus* spp. (n = 3/3)
Ampicillin	454 (30.8/89.4)	141 (9.6/97.2)	31 (2.1/96.9)	22 (1.5/95.7)	105 (7.1/98.1)	NA	0 [6]	1 (0.1/33.3)
Amoxycillin-Clavulanic acid	366 (24.9/72.0)	120 (8.2/82.8)	31 (2.1/96.9)	14 (0.9/60.9)	80 (5.4/74.8)	NA	NA	1 (0.1/33.3)
Trimethoprim-Sulfamethoxazole	354 (24.0/69.7)	90 (6.1/62.1)	17 (1.2/53.1)	9 (0.6/39.1)	56 (3.8/52.3)	21 (1.4/65.6)	NA	0 [6]
Ciprofloxacin	74 (5.0/14.6)	26 (1.8/17.9)	3 (0.2/9.4)	1 (0.1/4.3)	18 (1.2/16.8)	9 (0.6/28.1)	0 [6]	0 [6]
Chloramphenicol	103 (7.0/20.3)	36 (2.4/24.8)	5 (0.3/15.6)	4 (0.3/17.4)	18 (1.2/16.8)	4 (0.3/12.5)	0 [6]	0 [6]
Gentamicin	111 (7.5/21.9)	37 (2.5/25.5)	6 (0.4/18.8)	3 (0.2/13.0)	22 (1.5/20.6)	7 (0.5/21.9)	1 (0.1/33.3)	0 [6]
Amikacin	125 (8.5/24.6)	33 (2.2/22.8)	9 (0.6/28.1)	2 (0.1/8.7)	19 (1.3/17.8)	6 (0.4/18.8)	0 [6]	0 [6]
Ceftriaxone	92 (6.3/18.1)	42 (2.9/29.0)	4 (0.3/12.5)	4 (0.3/17.4)	31 (2.1/29.0)	NA	NA	0 [6]
Cefuroxime	366 (24.9/72.0)	92 (6.3/63.4)	28 (1.9/87.5)	8 (0.5/34.8)	83 (5.6/77.6)	NA	NA	1 (0.1/33.3)
Ceftazidime	55 (3.7/10.8)	30 (2.0/20.7)	1 (0.1/3.1)	3 (0.2/13.0)	7 (0.5/6.5)	13 (0.9/40.6)	0 [6]	0 [6]
Imipenem	41 (2.8/8.1)	19 (1.3/13.1)	9 (0.6/28.1)	1 (0.1/4.3)	27 (1.8/25.2)	2 (0.1/6.3)	0 [6]	0 [6]
Tetracycline	NA	NA	NA	NA	NA	9 (0.6/28.1)	0 [6]	NA
Piperacillin	NA	NA	NA	NA	NA	16 (1.1/50.0)	0 [6]	NA
Piperacillin-Tazobactam	NA	NA	NA	NA	NA	11 (0.7/34.4)	0 [6]	NA
Colistin	NA	NA	NA	NA	NA	NA	0 [6]	NA
ESBL-producing bacteria	36 (2.4/7.1)	16 (1.1/11.0)	1 (0.1/3.1)	1 (0.1/4.3)	3 (0.2/2.8)	NA	NA	0 [6]
Carbapenem-resistant bacteria	41 (2.8/8.1)	19 (1.3/13.1)	9 (0.6/28.1)	1 (0.1/4.3)	27 (1.8/25.2)	2 (0.1/6.3)	0 [6]	0 [6]
MDR	426 (28.9/83.9)	89 (6.0/61.4)	22 (1.5/68.8)	6 (0.4/26.1)	62 (4.2/57.9)	18 (1.2/56.3)	2 (0.1/66.7)	1 (0.1/33.3)
MDR^b^	350 (23.8/68.9)	54 (3.7/37.2)	12 (0.8/37.5)	4 (0.3/17.4)	32 (2.2/29.9)	18 (1.2/56.3)	2 (0.1/66.7)	1 (0.1/33.3)

^a^NA indicates that the antibiotic was not tested or was not relevant for the given organism

^b^MDR excluding ESBL-producing and carbapenem-resistant bacteria

^c^The two numbers in parentheses indicate number of colonized women and the number of isolates of each given species, respectively

**Table 2:** Vaginal colonization with antimicrobial drug-resistant potentially pathogenic gram-positive bacteria among study women in labor

Antibiotics^a^	Number of the 1472 study women who were colonized with bacteria resistant to one or more antibiotics (prevalence of colonization /proportion of isolates, in %)			
	*Enterococcus* spp. (n = 47/47)^c^	*S. aureus* (n = 117/121)	Group A *Streptococcus* (n = 3/3)	Group B *Streptococcus* (n = 3/3)
Trimethoprim-Sulfamethoxazole	NA	77 (5.2/63.6)	2 (0.1/66.7)	1 (0.1/33.3)
Ciprofloxacin	40 (2.7/85.1)	48 (3.3/39.7)	NA	NA
Chloramphenicol	19 (1.3/40.4)	26 (1.8/21.5)	0 [6]	1 (0.1/33.3)
Gentamicin	NA	38 (2.6/31.4)	NA	NA
Penicillin	18 (1.2/38.3)	93 (6.3/76.9)	0 [6]	1 (0.1/33.3)
Ampicillin	NA	NA	NA	1 (0.1/33.3)
Tetracycline	30 (2.0/63.8)	70 (4.8/57.9)	3 (0.2/100)	3 (0.2/100)
Erythromycin	30 (2.0/63.8)	89 (6.0/73.6)	2 (0.1/66.7)	3 (0.2/100)
Vancomycin	6 (0.4/12.8)	6 (0.4/5.0)	1 (0.1/33.3)	1 (0.1/33.3)
Ceftriaxone	NA	NA	0 [6]	0 [6]
Linezolid	8 (0.5/17.0)	NA	NA	NA
Oxacillin	37 (2.5/78.7)	85 (5.8/70.2)	1 (0.1/33.3)	1 (0.1/33.3)
MRSA	NA	85 (5.8/70.2)	NA	NA
VRSA/VRE	6 (0.4/12.8)	6 (0.4/5.0)	NA	NA
iMLSB-resistant *S. aureus*	NA	18 (1.2/14.9)	NA	NA
cMLSB-resistant *S. aureus*	NA	24 (1.6/19.8)	NA	NA
MDR	37 (2.5/78.7)	85 (5.8/70.2)	1 (0.1/33.3)	1 (0.1/33.3)
MDR^b^	31 (2.1/66.0)	0 [6]	1 (0.1/33.3)	1 (0.1/33.3)

^a^NA indicates that the information is not relevant for the given resistance pattern

^b^MDR excluding MRSA, VRSA, VRE, iMLSB- and cMLSB-resistant *S. aureus*

^c^The two numbers in parentheses indicate number of colonized women and the number of isolates of each given species, respectively

### Exposure factors

To identify potential risk factors for colonization with different AMR bacteria in the logistic regression models described below, we included the following exposure information, which were acquired during interviews with the mothers after they had given birth. PROM defined as rupture of membranes before the start of labour [25], parity, maternal level of education, maternal age, hospitalization during pregnancy, antenatal care attendance, ownership of domestic animals at home and socioeconomic status. As a measure of socioeconomic status, we used principal component analysis on an asset index that we generated by evaluating the woman’s access to or ownership of cupboards, radios, televisions, a mobile phone, refrigerator, motorcycle, car, ownership of a house, and presence of cemented walls, flushing toilet, and having three or more rooms in the house. Socioeconomic status was divided into 5 levels, where the poorest women were categorized as belonging to the 1st quintile while the richest were categorized as belonging in the 5th quintile.

#### Statistical analyses

The statistical analyses were done using Stata version 15.0 (StataCorp, College Station, Texas, USA). We obtained the overall prevalence of MDR colonization by dividing the number of women colonized with MDR bacteria by the total number of women enrolled into the study. To obtain the overall prevalence of resistance with clinically important bacteria such as MRSA, MLSB-resistant *S. aureus*, VRSA, VRE, ESBL and carbapenem-resistant bacteria, we divided the number of women colonized with such bacteria by the total number of enrolled women in the study. All proportions were reported with their respective 95% confidence intervals, which were calculated using the exact method.

We performed multivariable logistic regression analyses to explore the association between selected exposures (maternal age, maternal education, socioeconomic status, gravidity, number of antenatal visits, hospitalization during pregnancy, ownership of domestic animals) and our primary outcome of vaginal colonization with MDR bacteria and secondary outcomes including ESBL and MRSA bacteria. We chose to conduct exploratory multivariable analyses because the exposures selected potentially confounded one another.

We used the estat vif command in STATA to test the models for potential multicollinearity between the independent variables, as indicated by one or more variance inflation factor estimates being > 10. None of our models appeared to have potential multicollinearity issues.

## Results

### Bacterial isolates

A total of 1472 women in labour with an average age of 24.6 years (standard deviation: 4.9 years) were enrolled in the study. We obtained 1025 potentially pathogenic bacterial isolates from vaginal specimens from 955 (64.9%) of the women, including 1 isolate from 878, 2 from 69, and 3 from 3 women. The 1025 isolates represented 851 (83%) gram-negative and 174 (17%) gram-positive isolates, and included 508 (49.6%) *Escherichia coli*, 145 (14.1%) *Klebsiella pneumoniae*, 121 (11.8%) *Staphylococcus aureus*, 107 (10.4%) *Citrobacter* spp., 47 (4.6%) *Enterococcus* spp., 32 (3.1%) *Enterobacter* spp., 32 (3.1%) *Acinetobacter* spp., 23 (2.2%) *Klebsiella oxytoca*, 3 (0.3%) *Pseudomonas* spp., 3 (0.3%) group A *Streptococcus*, 3 (0.3%) group B *Streptococcus*, and 1 (0.1%) *Proteus* sp. Nine of the 955 specimens yielded more than one isolate for a given species. These included four specimens with two *E. coli* isolates, one specimen with two *K. pneumonia* isolates, and four specimens with two *S. aureus* isolates.

### Antimicrobial resistance profiles of the potentially pathogenic bacteria

Antimicrobial resistance results are listed in Tables 1 and 2. Of the 1472 women, 976 (66.3%; 95% CI 63.8%, 68.7%) were colonized with bacteria resistant to at least one of the first-line antibiotics used for treating severe neonatal infections in Uganda, including ampicillin and gentamicin.

### Colonization with ESBL-producing bacteria

Of the 1472 women, 57 (3.9%; 95% CI: 3.0%, 5.1%) were colonized with ESBL-producing bacteria, all of which were *Enterobacteriaceae* (Table 1). The 57 isolates recovered from these 57 women included 36 *E. coli*, 16 K*. pneumoniae,* 3 *Citrobacter* spp., 1 K*. oxytoca*, and 1 *Enterobacter* spp. Except for 1 *E. coli* and 1 K*. pneumoniae*, all these ESBL-producing *Enterobacteriaceae* isolates were MDR.

### Colonization with carbapenem-resistant bacteria

We found that 27 of the 1472 women (1.8%; 95% CI: 1.2%, 2.7%) were colonized with carbapenem-resistant bacteria. All the carbapenem-resistant bacteria were *Enterobacteriaceae* including: 10 *E. coli,* 7 *Citrobacter* spp., 5 K*. pneumoniae*, 3 *Enterobacter* spp., and 1 K*. oxytoca* isolates; except one carbapenem-resistant *Acinetobacter* spp. None of the *Pseudomonas* spp. strains were carbapenem-resistant. All the carbapenem-resistant bacteria were MDR.

### MRSA, MLSB, VRSA and VRE colonization

**Figure 1:**
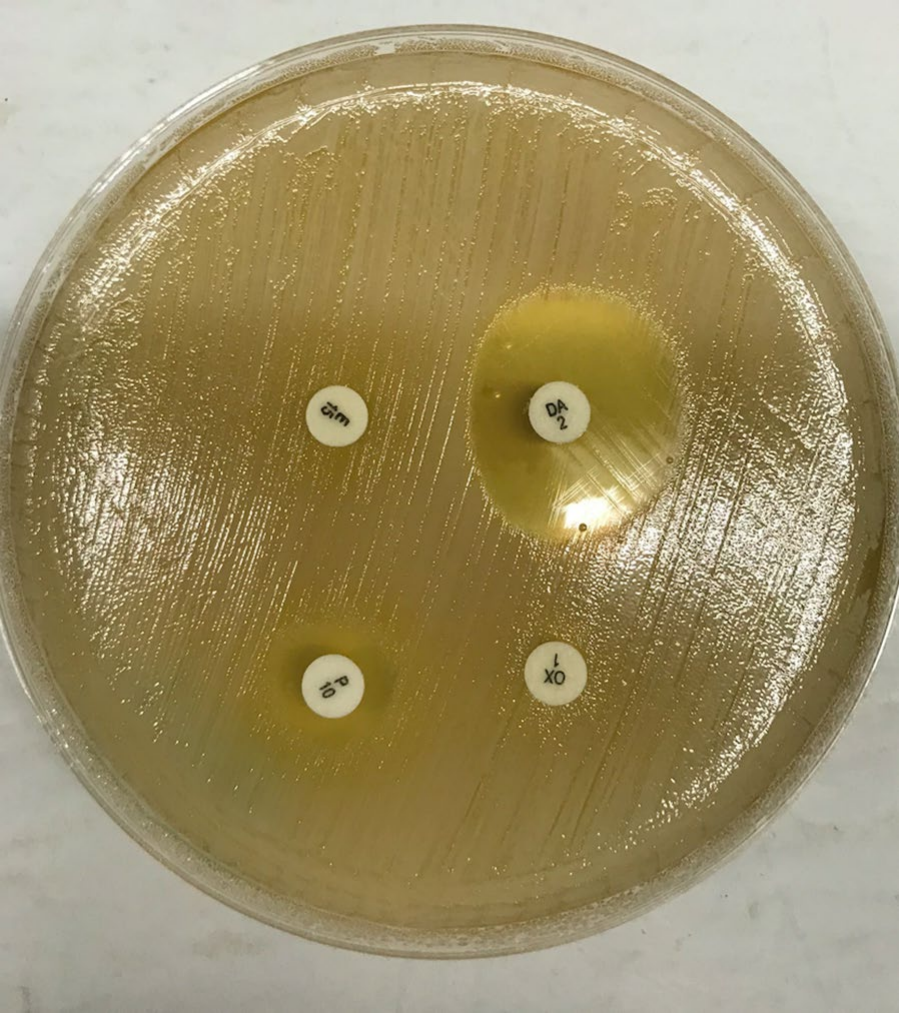
Example of a disk diffusion test for inducible clindamycin resistance in a *S. aureus.* isolate. Inducible clindamycin resistance is indicated by the flattened border between the clindamycin disk (top right) and the erythromycin disk (top left), resulting in a characteristic 'D'-shaped area cleared of *S. aureus.* around the clindamycin disk. The two lower disks were included to test the isolate for penicillin (left) and oxacillin (right) resistance.

We found that 117 of the 1472 women were colonized with *S. aureus.* Among these, 85 (5.8% [95% CI 4.6, 7.1]) were colonized with MRSA. Forty-five of 55 *S. aureus* isolates (81.8%) tested positive in our PCR-based MRSA assay, but of these only 2 (3.4%) were positive for the Panton-Valentine Leukocidin (PVL) virulence factor. The three other PVL positive isolates were from methicillin sensitive *S. aureus*. All MRSA isolates were MDR. Eighteen of the 1472 women (1.2% [95% CI 0.7%, 1.9%]) were colonized with induced macrolide lincosamide-streptogramin B (iMLSB)-resistant *S. aureus* while 24 (1.6% [95% CI 1.0%, 2.4%]) were colonized with constitutive MLSB (cMLSB)-resistant *S. aureus*. Fifteen of the 18 iMLSB-resistant *S. aureus* (83.3%) isolates were also MRSA. The proportion of women colonized with VRSA and VRE was both 0.4% (6/1472; 95% CI 0.1%, 0.9%). All 12 isolates from these VRE and VRSA colonization were MDR (Fig. 1).

### Colonization with MDR bacteria

We found that 750 (50.9%; 95% CI 48.4%, 53.5%) of the women were colonized with MDR bacteria (Tables 1 and 2). The majority of colonizations with MDR bacteria included *E. coli* (56.8% [426/750]), *K. pneumoniae* (11.9% [89/750]) and *S. aureus* (11.2% [84/750]). The distribution of characteristics of women colonized with MDR bacteria is shown in Table 3.

**Table 3:** Association between exposures and vaginal colonization with important antibiotic-resistant bacteria among study women in labor

Participant characteristic	No. of women (%), N = 1472	Adjusted odds ratio (95% CI) of being colonized with bacteria		
		ESBL-producing *Enterobacteriaceace*	MRSA	MDR
Mother's age				
20–24 years	587 (39.9)	1	1	1
< = 19 years	205 (13.9)	0.69 (0.26, 1.79)	1.26 (0.59, 2.68)	1.16 (0.82, 1.63)
25–29 years	454 (30.8)	1.32 (0.69, 2.54)	1.68 (0.93, 3.02)	1.27 (0.98, 1.65)
> = 30 years	226 (15.4)	0.91 (0.34, 2.43)	3.03 (1.51, 6.07)	1.56 (1.09, 2.24)
Education level				
Tertiary	130 (8.8)	1	1	1
Primary	488 (33.2)	1.15 (0.39, 3.33)	1.91 (0.74, 4.93)	1.31 (0.86, 1.98)
Secondary	854 (58.0)	1.08 (0.40, 2.89)	1.44 (0.58, 3.53)	1.42 (0.96, 2.09)
Wealth index				
Quintile5	290 (19.7)	1	1	1
Quintile1	489 (33.2)	0.82 (0.36, 1.85)	1.09. (0.54, 2.20)	0.82 (0.59, 1.13)
Quintile2	100 (6.8)	0.42 (0.09, 1.97)	0.85 (0.29, 2.51)	0.85 (0.53, 1.38)
Quintile3	298 (20.2)	0.86 (0.36, 2.09)	1.07 (0.50, 2.31)	0.86 (0.60, 1.23)
Quintile4	295 (20.0)	1.06 (0.45, 2.49)	0.97 (0.45, 2.06)	0.86 (0.61, 1.22)
Gravidity				
Primigravida	442 (30.0)	1	1	1
2–4 pregnancies	910 (61.8)	0.73 (0.37, 1.44)	0.84 (0.46, 1.55)	0.95 (0.73, 1.25)
5 or more pregnancies	120 (8.2)	0.68 (0.18, 2.59)	0.39 (0.13, 1.19)	0.62 (0.37, 1.02)
Hospitalization during pregnancy				
No	1387 (94.2)	1	1	1
Yes	710 (94.5)	1.35 (0.47, 3.33)	1.32 (0.55, 3.17)	0.99 (0.64, 1.55)
Antenatal visits				
Once	75 (5.1)	1	1	1
2–4 times	1259 (85.5)	0.53 (0.20, 1.41)-	0.78 (0.30, 2.02)	1.15 (0.71, 1.85)
5 or more times	138 (9.4)	0.28 (0.06, 1.26)-	1.29 (0.42, 3.95)	1.12 (0.63, 1.99)
Own domestic animals				
No	1355 (92.1)	1	1	1
Yes	117 (8.0)	0.16 (0.02, 1.27)	0. 89 (0.34, 2.35)	0. 77 (0.50, 1.20)

Even when omitting the clinically important AMR bacteria (ESBL-producers, carbapenem- and MLSB-resistant bacteria, MRSA, VRSA and VRE), as many as 506 (34.5%) of the women were colonized with MDR bacteria.

### Exposures associated with colonization with AMR bacteria

We found that being ≥ 30 years old was associated with an increased odds of being colonized with MRSA (adjusted OR: 3.03 [95% CI: 1.51, 6.07]) or MDR (adjusted OR: 1.56 [95% CI: 1.09, 2.24]) compared to 20–24 year olds. Wealth level was not associated with AMR colonization (Table 3).

## Discussion

In this study, we investigated the prevalence of vaginal colonization with clinically important AMR bacteria among women in labour at three primary health care facilities in and close to Kampala in central Uganda; and established exposures associated with AMR. The prevalence of colonization with MDR bacteria in our study was 46.6%, similar to those reported from Malawi and Ethiopia [26–28]. Although direct comparison of results between studies are complicated by the lack of consensus on how MDR should be defined, we used an internationally acknowledged MDR definition, making our findings comparable to those from similar studies [29, 30]. With the main exceptions of gentamicin, ampicillin and ceftriaxone, most of the antibiotics included in this study are not commonly used to treat serious bacterial infection in children in Uganda. Nevertheless, the antibiotic classes of all relevant first- and second-line antibiotics used in Uganda should be represented in this study.

The prevalence of women colonized with ESBL-producing bacteria in our study was low (4%). Our findings are similar to those reported from other comparable studies [31–34]. However, studies in similar settings such as Tanzania [35] and India [36] found a prevalence of ESBL vaginal colonization to be higher (15%) and a study in Bangladesh found a prevalence of women colonized with ESBL-producing bacteria to be 21% [37]. It is not clear why there is a large difference in prevalence of vaginal colonization with these ESBL-producing bacteria between these studies, but since ESBL encoding genes are easily spread between bacteria [38], it is possible that the differences in prevalence could be effects of differences in antibiotic use and, thereby, selective pressures between the study populations. The discrepancies may also be explained by the differences in microbiological methods, sampling error and study populations as the women we studied were low risk and averagely young women aged about 25 years.

The prevalence of colonization with carbapenem-resistant bacteria in our study was 2%, similar to that reported among Algerian pregnant women [39] and among women in labour at Brooklyn, New York Hospital [40]. These studies including ours suggest the importance to assess maternal colonization with carbapenem-resistant bacteria during labour as there is potential for vertical transmission of these bacteria to newborns.

Overall, the prevalence of colonization with MRSA was 5.8%, which is somewhat higher than that observed in other similar studies that reported a prevalence 0.5%-3.5% [41–47]. This may be explained by differences in geographical setting as well as differences in microbiological testing methods used in our study compared to other similar studies. MRSA is usually resistant to majority of beta-lactam drugs including penicillins, beta-lactamase inhibitors, cephalosporin and carbapenems. However, MRSA are sensitive to ceftaroline suggesting general overuse and/or misuse of antibiotics. Resistance to these drugs occurs because of acquisition of genes that encode drug-inactivating enzymes. However, a majority of the *S. aureus* isolates in our study were highly susceptible to vancomycin. Vancomycin is still the first line drug for treating MRSA infections in many countries, but it remains an expensive drug to be used in resource limited settings like Uganda.

The very low prevalence (0.4%) of VRSA in our study is similar to that observed (0.9%) in a South African cohort of pregnant women [47]. Relatively new anti-MRSA drugs (linezolid, tigecycline, daptomycin and quinupristin-dalfopristin) were introduced after the emergence of VRSA but they are expensive, especially in resource limited settings. Increased spread of MRSA and VRSA are of public health concern since they render treatment efforts with affordable beta lactam antibiotics futile. The prevalence of PVL positive MRSA was 0.3% in our study which is comparable to the 0.9% reported among Bangladeshi pregnant women [37].

In our study, we assessed for MLSB resistance also known as erythromycin-inducible clindamycin resistance. We observed a prevalence of vaginal colonization with erythromycin-inducible clindamycin resistance among study participants to be 1.2% similar to the 0.7% observed among pregnant women in an Egyptian study [48]. The majority of studies on vaginal colonization of pregnant women with MLSB resistant bacteria report such resistance among GBS compared to *S. aureus* in our study; hence making it difficult to directly compare our findings. We reported a low prevalence of 0.4% among study participants and our findings are similar to those of others [49]. However, our findings differ from those in Ethiopia and the discrepancy may be due to differences in antibiotic prescription and or antibiotic use, geographical settings, study populations and differences in VRE detection methods. Vaginal colonization of women in labour with VRE is of concern to the newborn in case of vertical transmission because it would greatly limit the options of effective treatment of serious infections, leading to poor clinical outcomes among the neonates.

Generally, unregulated access of antibiotics over the counter and their increased use in domestic and commercial animal farming contributes significantly to the antimicrobial resistance problem, also in resource limited settings [50]. Irrational use of antibiotics is a major public health problem globally and is associated with increase in antibiotic resistance [51]. A multi-site study conducted in Uganda found that 41% of antibiotics were issued over the counter without prescription [52]. This suggests that irrational use of antibiotics is very common in Uganda.

In the present study, we observed that women aged 30 or more years were more likely to be colonized by MDR or MRSA than 20–24 years old women. Our findings are similar to another study that found that Moroccan women who were older were more likely to be colonized with multi-drug resistant bacteria [53]. We tested exposures that other studies found to be associated with vaginal colonization [54–56]. Unlike those studies, we did not find substantial associations between colonization with antimicrobial resistant bacteria and exposures such as living with domestic animals, prior hospitalization or prior health care facility visits. One potential explanation could be the differences in geographical settings between the studies. Another potential explanation is the likely absence of power to study these exposures given our relatively wide confidence intervals.

We conducted a large study with 1472 women recruited from three health facilities in and close to Kampala. The study had some limitations. We did not perform molecular antimicrobial resistance assays except for MRSA (*mecA* PCR) to confirm the phenotypic resistance patterns we observed using the disk diffusion method. Given that the resistance phenotype of some bacteria may be conditional on the culture condition, we cannot rule out the possibility that we may have underestimated the prevalences of colonization with AMR bacteria in this study given we have only tested the isolates for resistance by using the disk diffusion method. By not performing anaerobe cultures, we probably missed colonization with some pathogenic bacteria that could contribute to severe infections in neonates, and it may have reduced our ability to identify a few facultative anaerobic bacteria that may be easier to identify when grown under anaerobic conditions [7]. Finally, our use of traditional biochemical tests to determine the species of each isolate may not always give accurate results. Consequently, we may have somewhat underestimated the true prevalence of some of the infections.

## Conclusion

The prevalence of vaginal colonization with potentially pathogenic MDR and other clinically important AMR bacteria among HIV-negative women in labour at three primary health care facilities was high. The finding of extensive colonization with multidrug-resistant bacteria including ESBL-producing *Enterobacteriaceace*, carbapenem-resistant bacteria, methicillin-resistant *S. aureus*, erythromycin-inducible clindamycin resistant-*S. aureus*, vancomycin-resistant *S. aureus* and vancomycin-resistant *Enterococc*i in our study raises a question whether we should conduct routine screening of pregnant women or exposed newborns for carriage/colonization. However, screening women during antenatal would be expensive and identifying the exposed infants would increase on the existing huge workload for health workers. Our findings have implications for possible prophylactic treatment to pregnant women colonized with such multidrug resistant bacteria, the prevention and management of early on-set neonatal sepsis including providing local data to guide choice of antibiotics for treating early-onset neonatal sepsis and vaccine development in similar settings. There is need to investigate whether there is vertical transmission of these multidrug-resistant bacteria to the babies.

## Data Availability

Datasets used during the current study can be obtained through a reasonable request from the principle investigator of the Chlorhexidine Trial (VN) nankabirwav@gmail.com and the corresponding author.
